# Silent left ventricular apical ballooning and Takotsubo cardiomyopathy in an Australian intensive care unit

**DOI:** 10.1002/ehf2.12517

**Published:** 2019-09-26

**Authors:** Alexandra C. Rowell, Wade G. Stedman, Pierre F. Janin, Naomi Diel, Michael R. Ward, Sharon M. Kay, Anthony Delaney, Gemma A. Figtree

**Affiliations:** ^1^ Kolling Institute University of Sydney Sydney NSW Australia; ^2^ Malcolm Fisher Department of Intensive Care Medicine Royal North Shore Hospital St Leonards NSW Australia; ^3^ Department of Cardiology Royal North Shore Hospital St Leonards NSW 2065 Australia; ^4^ Division of Critical Care, The George Institute for Global Health UNSW Sydney Australia

**Keywords:** Intensive care, Takotsubo cardiomyopathy, Inotropes, Adrenergic, Echocardiography

## Abstract

**Aims:**

Recent reports have shown a high incidence of silent left ventricular apical ballooning (LVAB) in the intensive care unit (ICU) setting with potential implications for safe use of inotropes and vasopressors. We examined the incidence, predictors, and associated outcomes of LVAB in patients in a contemporary tertiary Australian ICU.

**Methods and results:**

In a prospective cohort study, patients were screened within 24 h of admission to the ICU and enrolled if they were deemed critically unwell based on mechanical ventilation, administration of >5 mg/min of noradrenaline, or need for renal replacement therapy. Exclusion criteria were a primary diagnosis of Takotsubo cardiomyopathy, admission to ICU after cardiac surgery, or with acute myocardial infarction or heart failure. Echocardiography was performed, and the presence/absence of LVAB was documented. A total of 116 patients were enrolled of whom four had LVAB (3.5%, 95% confidence interval 0.9–8.6%). Female sex was the only baseline demographic or clinical characteristic associated with incident LVAB. Medical history, ICU admission indication, and choice of inotropes were not associated with increased risk. Patients with LVAB had no deaths and had similar lengths of ICU and hospital stay compared with patients with no LVAB.

**Conclusions:**

The incidence of silent LVAB suggestive of TC was substantially lower in this study than recently reported in other international ICU settings. We did not observe a suggestion of worse outcomes. A larger, multi‐centre study, prospectively screening for LVAB may help understand any variation between centres and regions, with important implications for ICU management.

## Introduction

Takotsubo cardiomyopathy (TC) is a syndrome of acute reversible left ventricular dysfunction, classically with apical ballooning (LVAB) and basal preservation, which can present with chest pain and ischaemic electrocardiograph changes and mimic acute coronary syndrome.^1^ The syndrome is often precipitated by severe psychological or physical stress, more commonly in post‐menopausal women.^2^, ^3^ Whilst the exact cause of the condition is unknown, it is widely accepted that it is related to high levels of circulating catecholamines and dysregulated adrenergic signalling at the receptor level.^3^, ^4^, ^5^, ^6^ Recent studies have reported a variable, but surprisingly high, incidence of undiagnosed LVAB in some intensive care settings. An incidence of 28% in medical ICU patients was reported in a South Korean study that screened 92 consecutive patients by echocardiography^5^ and 5.6% of the medical ICU patients who required echocardiography for clinical and haemodynamic reasons.^7^ Given this considerable number of patients, the association with worse clinical outcomes,^5^, ^7^ and the potential harm caused by use of certain inotropes,^4^ we set out to examine the incidence of LVAB in patients admitted to a tertiary Australian ICU, and if significant, to identify significant predictors of risk and association with outcomes.

## Methods

We performed a prospective cohort study in a tertiary ICU at Royal North Shore Hospital in Sydney. Patients (*n* = 116) were enrolled between January 2014 and June 2015, within 48 h of admission to the ICU. Patients were eligible for enrolment if they were ≥ 18 years of age and critically unwell defined as requiring at least one of the following: more than 5 μg/min of noradrenaline for more than 4 h, mechanical ventilation, and renal replacement therapy. Patients were excluded if they had an admission diagnosis of TC, if they were admitted to the ICU following cardiac surgery, or with a primary diagnosis of an acute myocardial infarction or heart failure. Study recruitment depended on the availability of the study team. Consent was obtained from the patient or when not practicable by the next of kin. The study complies with the principles outlined in the Declaration of Helsinki, and the process was approved by our local HREC (ref. LNR/13/HAWKE/249).

Baseline data included cardiac history, ICU admission details, and known risk factors for TC, including post‐menopausal status. Echocardiography was performed and independently reviewed in a blinded fashion by a cardiologist and an intensive care specialist with a Diploma in Diagnostic Ultrasound. Standard echocardiographic reporting was performed with particular consideration of the pattern of any left ventricular systolic abnormality and its consistency with LVAB/TC. Mortality and length of stay data were collected at a later date using the hospital information system.

Data were reported using numbers and proportions for categorical data, mean, and standard deviation for normally distributed continuous data and median and interquartile range (IQR) for non‐normally distributed data. Baseline data and mortality in the groups were compared using the Fisher's exact test and length of stay using the Mann–Whitney test. The number of cases and estimate of the proportion of patients with the condition were reported with exact 95% confidence intervals.

## Results

In total, 116 patients were enrolled into the study. Of these patients, four (3.5%, 95% confidence interval 0.9–8.6%) were found to have LVAB, in a Takotsubo‐like pattern on echocardiography. One of the four LVAB patients had an angiogram confirming no significant coronary artery disease. The remainder with LVAB and no angiography were assumed to have TC based on the non‐coronary distribution of the wall‐motion abnormalities and low clinical suspicion of coronary artery disease.

Baseline demographics and ICU admission characteristics are shown in *Table* 1. Female sex was the only significant demographic or clinical feature to be associated with the risk of LVAB. All four of the four patients with LVAB were women (vs. 38% of the no LVAB group; *P* = 0.025). The median age of LVAB patients was 66 years (IQR 53.5–78.4) [versus 58 years in no LVAB (IQR 44.9–72.6); *P* = 0.37]. Within the limitations of this study, there were no significant differences in the cardiac risk factors between the groups, use of beta‐blockers, or history of asthma or chronic obstructive pulmonary disease. Two of the four LVAB patients were post‐menopausal, similar in proportion to the female non‐LVAB group (60%).

**Table 1:** Baseline demographics and ICU admission characteristics

	No Takotsubo (112)	Takotsubo (4)	*P*‐value
Age, median (IQR)	58 (44.9–72.6)	66 (53.5–78.4)	0.32
Female, *n* (%)	43 (38.4%)	4 (100%)	0.03
Medical history			
History of IHD, *n* (%)	16 (14.3%)	1 (25%)	0.47
Beta‐blocker use, *n* (%)	22 (20%)	0 (0%)	0.99
Hypercholesterolaemia, *n* (%)	31 (28%)	0 (0%)	0.57
Diabetes, *n* (%)	17 (15%)	0 (0%)	0.99
Hypertension, *n* (%)	45 (40%)	0 (0%)	0.16
Smoker (%)	32 (29%)	1 (25%)	0.99
Post‐menopause if female, *n* (%)	26 (60%)	2 (50%)	0.99
Asthma/COPD, *n* (%)	15 (13%)	0 (0%)	0.99
Beta‐agonist use, *n* (%)	11 (10%)	0 (0%)	0.99
ICU primary diagnosis			
Respiratory, *n* (%)	13 (12%)	0 (0%)	
Cardiovascular, *n* (%)	4 (4%)	0 (0%)	
Sepsis, *n* (%)	29 (26%)	2 (50%)	
Cardiac arrest, *n* (%)	3 (3%)	0 (0%)	
Neurological, *n* (%)	26 (23%)	1 (25%)	0.73
Acute kidney injury, *n* (%)	7 (6%)	0 (0%)	
Surgical admission, *n* (%)	22 (20%)	0 (0%)	
Other, *n* (%)	8 (7%)	1 (25%)	
Inotrope requirements			
Noradrenaline, *n* (%)	82 (73%)	4 (100%)	0.57
Dose (μg/h), median (IQR)	486 (300–1000)	845 (584–1582)	0.16
Dobutamine, *n* (%)	5 (4%)	1 (25%)	0.19
Adrenaline, *n* (%)	5 (4%)	0 (0%)	0.99
Milrinone, *n* (%)	1 (1%)	2 (50%)	0.003
Levosimendan, *n* (%)	1 (1%)	0 (0%)	0.99
Continuous renal replacement therapy, *n* (%)	16 (14%)	0 (0%)	0.99
Invasive ventilation, *n* (%)	95 (85%)	3 (75%)	0.50
Non‐invasive ventilation, *n* (%)	12 (11%)	0 (0%)	0.99
FiO_2_, mean (SD)	0.496 (0.207)	0.475 (0.171)	0.84

COPD, chronic obstructive pulmonary disease; FiO_2_, fraction of inspired oxygen; ICU, intensive care unit; IHD, ischaemic heart disease; IQR, interquartile range.

The most common admission diagnosis in both groups was sepsis followed by a neurological diagnosis. Of the patients with a positive echocardiogram for LVAB, two were admitted with sepsis, one with a subarachnoid haemorrhage and the other with gastroenteritis. There were no significant associations between the use of specific inotropes or their dose and the incidence of LVAB.

Measures reflecting patient outcome are shown in *Table* 2. The median troponin in the LVAB group was 964 ng/L but varied substantially (6–15 401 ng/L). The median for the non‐LVAB group was 69 ng/L (IQR: 10–1413 ng/L). Lateral electrocardiograph changes were more frequent in the LVAB group (*P* = 0.041) but not significantly different in other territories. ICU and hospital mortality were zero in the LVAB group compared with 17% ICU mortality and 23% in‐hospital mortality in the non‐LVAB group, but the difference was not significant as a result of the low sample size in the LVAB group. Length of ICU and hospital stay were almost identical in the two groups.

**Table 2:** Cardiac markers, mortality, and length of stay in patients with no LVAB, compared with those with LVAB

	No LVAB	LVAB	*P*‐value
Troponin I (ng/L), median (IQR)	69 (10–1412.5)	962.5 (8–8658)	0.84
ECG changes at enrolment, *n* (%)			
Anterior	10 (9%)	0 (0%)	0.99
Inferior	7 (7%)	1 (25%)	0.26
Lateral	8 (8%)	2 (50%)	0.04
LBBB	3 (3%)	0 (0%)	0.99
ICU mortality, *n* (%)	19 (17%)	0 (0%)	0.99
Hospital mortality, *n* (%)	26 (23%)	0 (0%)	0.57
ICU length of stay, median days (IQR)	6 (3–12)	6 (4–22.5)	0.98
Hospital length of stay, median days after ICU admission (IQR)	16 (8–31.5)	15 (9–32.5)	0.87

ECG, electrocardiograph; ICU, intensive care unit; IQR, interquartile range; LBBB, left bundle branch block; LVAB, left ventricular apical ballooning.

## Discussion

By using echocardiographic screening in this single‐centre, contemporary Australian ICU setting, we found the incidence of TC/LVAB to be substantially lower than previous international studies at 3.5%. Furthermore, there was no significant association or trend towards worse outcome in those few individuals who did suffer from TC.

To our knowledge, this is the largest study performing echocardiographic screening for silent LVAB in the ICU population. Park *et al*. screened 92 patients using a similar approach and reported a 28% incidence of LVAB. Whilst Oras *et al*.^7^ included >1000 patients finding an incidence of ~5%, this was a retrospective study of those having echocardiography for clinical indications and is thus subject to significant selection bias. The lower incidence of LVAB in our study is perhaps even more surprising given our inclusion of neurosurgical patients, expected to have a higher incidence of LVAB due to the association with subarachnoid haemorrhage. The higher incidence demonstrated in the South Korean study may result from racial differences in susceptibility^8^ or from standard choice of inotropes. Whilst there was no significant difference between the groups in adrenaline dose in our study, and none of the patients in the LVAB group were receiving adrenaline, there was minimal use in the whole patient cohort. Differences in the choice of inotropic and vasopressor agents may account for the observed difference with other studies.

We used LVAB as surrogate for TC, where coronary angiography was deemed inappropriate given the low clinical suspicion for myocardial infarction or ischaemia to be the cause of the silent LV dysfunction. This provides a potential limitation to the study, as formal coronary angiography is part of the diagnostic criteria for TC.^1^ Although this may be considered a weakness of the study, demonstration of culprit coronary artery lesion would only decrease the number of true TC cases further.

In conclusion, we performed the largest echocardiographic screening study of TC in a contemporary ICU and found the incident diagnosis of TC to be substantially lower than previously described. The surprisingly low numbers of TC patients limited our power to evaluate the relationship between type of inotrope and development of this condition. However, the apparent variation compared with the higher incidence reported in other international centres points to the importance of a large, international, prospective screening study for LVAB, to further unravel potential contributors and management implications.

## Conflict of interest

None declared.

## Funding

GF was supported by the National Health and Medical Research council (APP1135920) and Heart Research Australia.
